# Editorial: Case reports in tumor genomics and ctDNA in precision medicine

**DOI:** 10.3389/fmed.2025.1724304

**Published:** 2025-11-14

**Authors:** Li Chen, Alice Chen

**Affiliations:** 1 Frederick National Laboratory for Cancer Research, Frederick, MD, United States; 2 AP Chen Consultant, Potomac, MD, United States

**Keywords:** ctDNA, precision medicine, genomic testing, variants of unknown clinical significance, oncology

The study of tumor genomics and circulating tumor DNA (ctDNA) has opened new avenues in cancer research, diagnosis, and treatment. When analyzing tumor samples or ctDNA from patients, researchers often come across genetic variations that are classified as variants of unknown significance (VUS). These variants pose a significant challenge as their clinical implications and impact on patient care remain uncertain. Patients with tumor genomics or ctDNA and VUS are individuals whose genetic profiles display specific genetic alterations that have not been definitively classified as pathognomonic, i.e., there is rare or insufficient evidence. These variants may include single-nucleotide changes, insertions, deletions, or other complex genetic rearrangements. Understanding the clinical significance of these variants is crucial for determining appropriate treatment strategies, as certain variants may impact disease prognosis, therapy response, and hereditary cancer risk assessment.

The collection of works for this Research Topic includes four case reports articles on tumor genomics or ctDNA in precision medicine, covering different types of variants on different cancer histologies. Xiong and Xia reported one case with ALK-positive mutation lung adenocarcinoma in which the patient achieved a prolonged progression-free survival after undergoing precise pleural effusion NGS and receiving combined bevacizumab treatment following multiple-line ALK-TKI resistance. Chang et al. identified a novel missense mutation in *SRRM2* through whole exome sequencing and Sanger sequencing in a patient who exhibited similar characteristics of previously reported cases of SRRM2-associated neurodevelopmental disorders, which expanded the spectrum of known mutations in SRRM2. Jin et al. discussed a rare case of an intracranial tumor with CIC-NUTM1 fusion and provided a literature review for such cases. Finally, Troullioud Lucas et al. reported a first case of Wilms tumor in an individual with Dias-Logan syndrome, in which a *de novo* likely pathogenic germline variant of *BCL11A* was detected.

The case reports in this Research Topic catalog these variants and their potential impact on patient treatment. It will benefit clinicians to optimize patient care and facilitate precision oncology approaches that target specific genetic alterations for improved outcomes in cancer patients. In conclusion, we thank all authors who contributed to the development of the articles and the reviewers for their valuable comments. We also encourage continued reporting of VUS that have clinical impact as these case reports contribute to the ultimate patient care.

